# An RNA ligase partner for the prokaryotic protein-only RNase P: insights into the functional diversity of RNase P from genome mining

**DOI:** 10.1128/mbio.00449-25

**Published:** 2025-04-29

**Authors:** Rekha Seshadri, Venkat Gopalan

**Affiliations:** 1 DOE Joint Genome Institute, Lawrence Berkeley National Laboratory 1666, Berkeley, California, USA; 2 Department of Chemistry and Biochemistry, The Ohio State University 201912 , Columbus, Ohio, USA; 3 Center for RNA Biology, The Ohio State University 2647, Columbus, Ohio, USA; Eunice Kennedy Shriver National Institute of Child Health and Human Development, Bethesda, Maryland, USA

**Keywords:** RNA ligase, HARP, tRNA biogenesis

## Abstract

RNase P can use either an RNA- or a protein-based active site to catalyze 5′-maturation of transfer RNAs (tRNAs). This distinctive attribute in the biocatalytic repertoire raises questions about the underlying evolutionary driving forces, especially if each variant somehow affords a selective advantage under certain conditions. Upon mining all publicly available prokaryotic genomes and examining gene co-occurrence, we discovered that an RNA ligase with circularization activity was significantly overrepresented in genomes that contain the protein form of RNase P. This unexpected linkage inspires testable ideas to understand the bases for scenarios that might favor RNase P variants of different architectures/make-up.

## OPINION/HYPOTHESIS

The maturation of tRNAs entails many processing and modification steps (1). Excision of the 5′ leader in precursor (pre) tRNAs is performed by RNase P. Isolation and characterization of bacterial RNase P revealed that the enzyme is a ribonucleoprotein (RNP) composed of a catalytic RNA and a protein cofactor (2). Subsequently, archaeal and eukaryotic RNase P were also shown to be RNPs powered by an RNA catalyst and aided by multiple protein subunits (3, 4). The discovery of a ribozyme in RNase P, along with examples of other catalytic RNAs, overturned the dogma that only proteins can function as biocatalysts, but it also spawned the notion that all RNase P variants must contain a catalytic RNA (5). This idea was disproved by the finding of RNA-free, protein-only RNase P (PRORP) in eukaryotes, and later of a bacterial/archaeal protein-only variant that was named homolog of *Aquifex* RNase P (HARP) (6–8). Unlike PRORPs that have so far not been found in the same subcellular compartment as the RNP version, HARP is present in some bacteria and archaea that also have the RNP form of RNase P (6–8). While the use of RNA- and protein-based active sites in RNase P to catalyze pre-tRNA cleavage represents a remarkable example of convergent evolution, the driving forces for and the payoffs from having these two guises in bacteria and archaea remain a mystery (8–10). Here, we sought to gain insights into the function of HARP inspired by the emerging picture.

The conspicuous absence of the bacterial RNase P RNP in members of Aquificaceae (e.g., *Aquifex aeolicus*) prompted a biochemical purification that culminated in the identification of the 23-kDa protein that was christened HARP (7). In *A. aeolicus*, HARP appears to be solely responsible for tRNA 5′ maturation and attests to its essentiality. However, HARP gene knockouts in the euryarchaeotes *Haloferax volcanii* and *Methanosarcina mazei* had no effect on growth under standard and select stress conditions (salt, temperature, nitrogen limitation) tested (11). By contrast, repression of the catalytic RNase P RNA in *H. volcanii* led to a growth lag and impaired tRNA processing (12). These findings are consistent with the idea that the RNP form of RNase P is the principal contributor to tRNA 5′ processing in these archaea, and by extension, likely in other euryarchaeal relatives where the RNP form and HARP are both present.

Other observations lend further intrigue to HARP’s function. First, the turnover number (*k*_cat_) of HARP is one to two orders of magnitude lower than the bacterial/archaeal RNP forms (7, 10, 13, 14). Also, recombinant *Hydrogenobacter thermophilus* (*Hth*, a thermophilic bacterium) HARP was found to cleave pre-tRNAs containing a 3′-CCA ~10-fold slower than those without (15). Since nearly one-fifth of *Hth* tRNAs have a genomically encoded 3′-CCA, HARP would not be optimal for 5′ processing of the entire pre-tRNA suite. Despite the caveats of *in vitro* assays, the documented weak activity and substrate specificity of HARP prompts the question of its ability to support robust growth. The counter perspectives are that HARP must be adequate in *A. aeolicus* (that lacks the RNP form) and that conditionally lethal genetic defects in *Escherichia coli* and *Saccharomyces cerevisiae* RNase P were complemented by HARP, albeit supporting slower growth than the parental strains (7). Moreover, if HARP functions only as a safeguard for situations where the RNP fails, its pre-tRNA cleavage activity need not be on par with the RNP variant. Second, the expectation that HARP (23 kDa) might offer a low-cost cellular alternative to the large, six-subunit RNP form (~200 kDa) is partly upended by the finding that HARP forms large oligomeric assemblies and requires at least a tetramer to support pre-tRNA cleavage (8, 10, 16). Third, four recombinant bacterial HARPs (from *Alkalilimnicola ehrlichii, Halorhodospira halophila, Thioalkalivibrio nitratrireducens,* and *Methylacidiphilum infernorum*) formed the expected oligomers but failed to process pre-tRNAs (17). Perhaps, pre-tRNA cleavage by some HARPs reflects a secondary activity (9–11) and is a trait not shared by all members. Last, phylogenetic analysis revealed that there is a patchy distribution of HARP at the class and genus level in many cases (9). For example, only 9 of 20 genomes in the genus *Haloarcula* possess a HARP gene.

While the above findings collectively support the idea that the RNP form of RNase P is the ancestral version that is near-universally tasked with tRNA biogenesis, it is unclear what factors shaped the use of these two divergent scaffolds for different or complementary cellular functions. To identify potential specialized roles for HARP, we mined all the publicly available prokaryotic genomes, examined gene co-occurrence networks, and made a surprising discovery.

Querying over a million prokaryotic genomes, we identified only 573 archaeal and 178 bacterial instances of HARP (PF09745) (Fig. S1 and S2; Table S1; see supplemental material for a description of the methods). A closer examination of the taxonomic groups and environmental metadata (isolation source) revealed that HARP is encoded predominantly by organisms residing in harsh or extreme environmental conditions, particularly high salinity, alkalinity, or temperature (Fig. S1; Table S1). Within archaea, HARP is found in various genera primarily within the orders *Halobacteriales, Methanosarcinales, Archaeaoglobales, Thermococcales, Methanococcales*, and *Methanobacteriales* (Fig. S2; Table S1); nearly 70% are from the *Halobacteriota* phylum, exemplifying the HARP-salinity connection. Within bacteria, occurrence is highly restricted to a few genera within the orders *Aquificales, Ectothiorhodospirales* (*Thioalkalivibrio* spp.), *Nitrococcales* (*Halorhodospira* spp.*, Aquisalimonas* spp.)*, Methylococcales (Methylocaldum* spp.), and T*hermodesulfovibrionales*. Isolation sources for these taxa are chiefly salterns, hydrothermal vents, and thermal springs (Fig. S1; Table S1).

**Fig 1:**
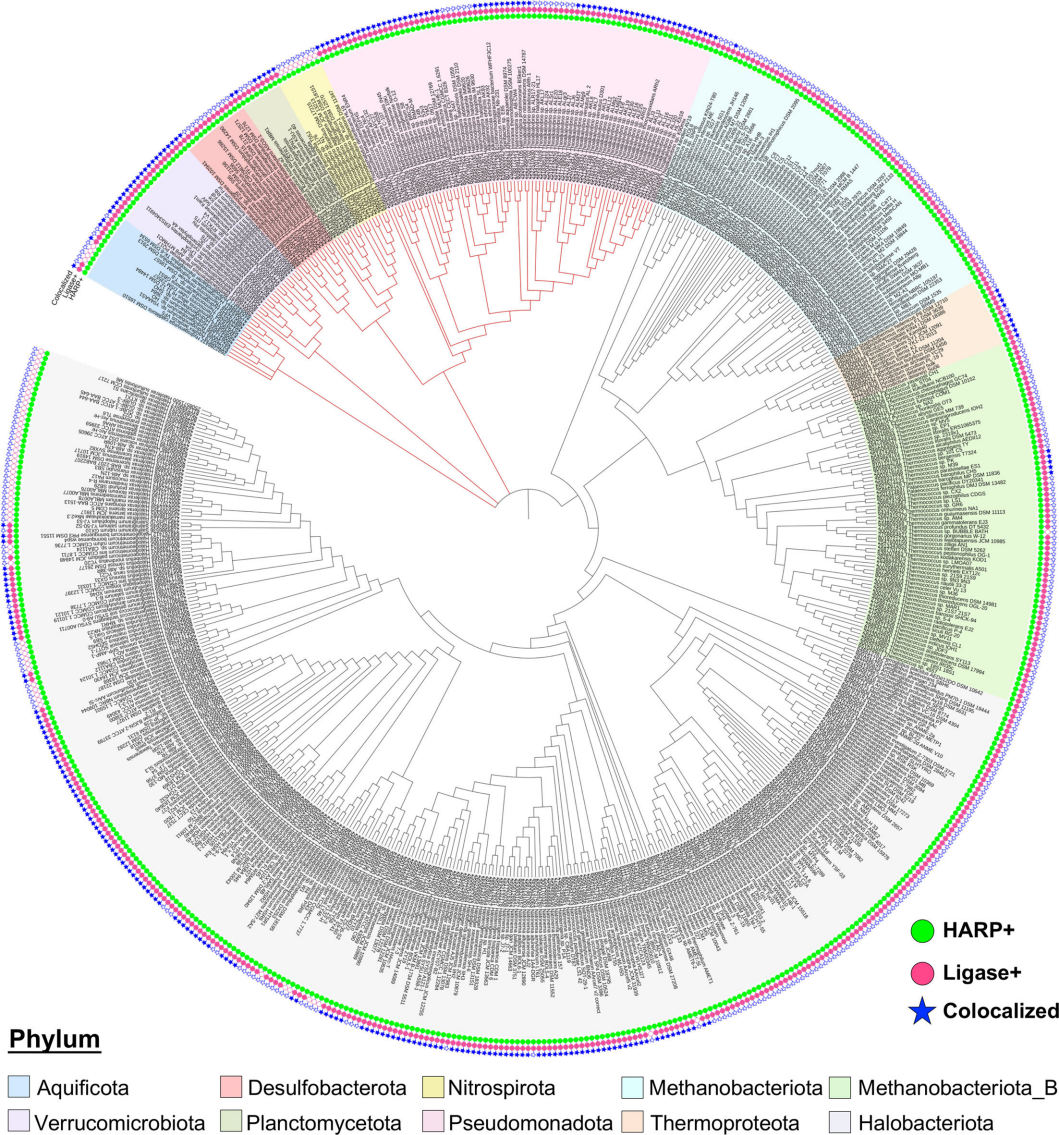
Phylogram of HARP-containing genomes of Bacteria (red branches) and Archaea (black branches). Tree labels are colored based on phylum. Concentric circles around the tree starting with the innermost are HARP-positive genomes, ligase-positive genomes, and co-localized HARP with ligase. The tree was visualized using iToL (22).

To gain insights into the selective pressures or conditions that may have led to a HARP-positive genotype, we compared archaeal HARP-positive *versus* HARP-negative genomes to identify any significantly over- or under-represented genes that may correlate with the positive/negative genotype (see supplemental methods). We found significantly overrepresented in the HARP-positive genomes an RNA ligase (Rnl, PF18330, TIGR01209), which is a member of the nucleotidyl-transferase domain superfamily that includes mRNA capping enzymes. This ligase in *Pyrococcus abyssi* was shown to exist as a homodimer and perform ATP-dependent circularization of various RNA substrates (18–20) and is classified as Rnl3 based on its structure and substrate specificity (there are six Rnl members, Rnl 1 to 6) (21). Cross-referencing ligase (Rnl3)-positive against the complete set of 751 HARP-positive genomes, we found that 665 (501 archaeal + 164 bacterial) or 89% of HARP-positive genomes are also ligase-positive (Fig. 1; Table S1 depicts this co-occurrence). Interestingly, all ligase-positive genomes are HARP-positive. Therefore, mirroring HARP, the distribution of RNA ligase is also highly restricted to the same small subset of archaea and bacteria from extreme environments.

Further scrutiny of the HARP-positive/ligase-positive genomes revealed that the RNA ligase is co-localized with the HARP gene (abutting or within very short distance [<5 kb] in a third of archaeal (163/501) and 80% of bacterial (127/164) cases. The position of the ligase is typically bidirectional relative to HARP. Only 10 instances of co-linear or tandem gene orientation occur in various archaeal genera within the order *Sulfolobales* and in one bacterium (*Desulfonatronospira thiodismutans*) (Table S1). This intriguing observation suggests a nexus between these previously unrelated activities and stimulates hypotheses (see below) on how these two enzymes may work in tandem or independently under specific environmental/physiological conditions.

The HARP-RNA ligase linkage provides an opportunity to consider a few ideas related to HARP’s function. First, while we cannot discount the possibility of concomitant selection pressures that led to independent retention of these two activities in harsh milieus (i.e., no direct functional cooperation), we inferred a functional link between HARP and RNA ligase (Rnl3) based on their co-occurrence in prokaryotes, especially their proximity in many cases. For instance, it is conceivable that the enhanced physicochemical stability expected of the oligomeric HARP, rather than the RNA-centered RNP catalyst, tilts the choice in HARP’s favor in extreme environments (e.g., catalytic RNase P RNA degradation under alkaline pH). Likewise, if these exacting conditions promote cellular RNA breakdown, the RNA ligase could provide a safety net through efficient RNA repair. Although we recognize that the HARP-RNA ligase co-occurrence could be fortuitous, the coincident patchy distribution of these two enzymes in ~700 bacterial and archaeal species seems unlikely to have resulted merely by chance.

Second, HARP is a member of the PIN superfamily of exo-/endo-nucleases, and it has been specifically placed structurally into the PIN_5 cluster of the group VapC (virulence-associated protein C) (23). Since one-fifth of this PIN superfamily are members of toxin-antitoxin (TA) systems, one possibility is that HARP and RNA ligase constitute a TA pair with HARP’s nucleolytic activity rendering it a toxin. Some TA systems are composed of two adjacent genes, as is the case with HARP and RNA ligase in some prokaryotes (Table S1). A stable TA complex keeps the toxin’s activity in check, with stress conditions or specific cues triggering antitoxin degradation and toxin activation. A corollary is that either depletion of the antitoxin or overexpression of the toxin without its partner antitoxin would be deleterious to growth. Indeed, expression of tRNA^fMet^-inactivating *Mycobacterium tuberculosis* VapC toxins in *M. smegmatis* led to severe growth inhibition (24), which could be rescued by co-expression of the corresponding antitoxin. Since the knockout of the RNA ligase (putative antitoxin) in *Thermococcus kodakarensis* (*Tko*)*,* a hyperthermophilic archaeon, did not elicit a growth defect (25), the HARP-RNA ligase pair is unlikely to function as a TA system, at least in *Tko*. However, *Tko* may not be the best model in this regard, because its HARP and ligase are not co-localized and therefore may not be subject to coordinate regulation.

**Fig 2:**
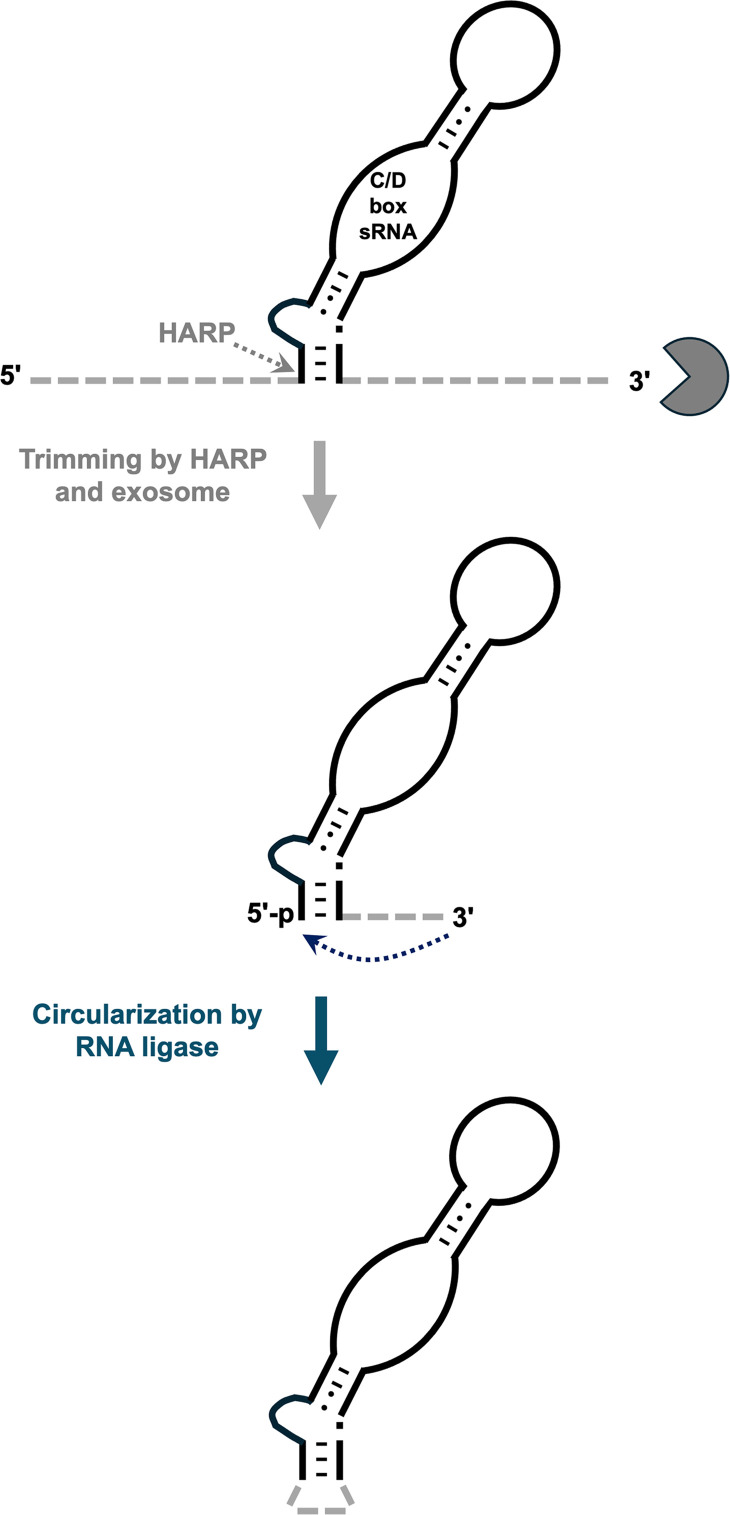
Proposal for the coupled action of HARP and RNA ligase that converts a C/D box RNA precursor into a final, mature circularized version. An arbitrary length was chosen to depict the 3′ trailer that is ligated by RNA ligase. Adapted from the biogenesis model presented in reference 30.

Finally, we sought clues to HARP’s function by focusing on the biological roles of the RNA ligase. Immunodepletion of the *P. abysii* RNA ligase followed by RNA-seq revealed that the enzyme plays a role in circularizing RNAs, especially select C/D box small nucleolar (sno) RNAs (26); this finding was independently confirmed when knockout of the RNA ligase in *Tko* impaired circularization of C/D box RNAs (25). By exploiting complementarity with substrates (e.g., rRNAs, tRNAs), C/D box snoRNAs (as part of an RNP in archaea and eukaryotes) guide site-specific 2′-O-methylation of their targets by the fibrillarin methyltransferase (27, 28). Thus, these snoRNAs play an essential role in proper ribosome assembly and translation. Circularization of C/D box RNAs through covalent linkage of the native 5′ and 3′ termini is predicted to enhance thermostability (29). An in-depth analysis of the genomic context of the C/D box small RNAs in six different archaea revealed that only 20% are likely to be transcribed from independent promoters and that the majority are co-transcribed with neighboring genes (27, 30). In either case, C/D box RNAs undergo maturation steps that entail nucleolytic trimming of their termini (27). Hence, as a testable hypothesis, we propose that HARP catalyzes 5′ maturation of these RNAs with other nucleases that execute 3′-trimming and set the stage for circularization by the RNA ligase (Fig. 2).

Another idea stems from the growing appreciation of diverse, non-canonical 5′ caps in prokaryotes and eukaryotes (i.e., NAD, ADP) (31). Inhibition of RNase P activity in *S. cerevisiae* was demonstrated to cause a build-up of 5′-methylated guanosine caps at the 5′-termini of pre-tRNAs (32), an observation consistent with the notion that caps may protect pre-tRNAs from exonucleolytic degradation when canonical 5′-maturation is impaired (32). This finding, together with the recent report that Rnl3 in the thermophilic archaeon *Paleococcus pacificus* exhibits strong substrate adenylation but weak ligation activity (21), prompts our proposal that specific stress conditions might promote 5′-adenylation of pre-tRNAs and that HARP functions post-stress to remove the 5′-adenylated pre-tRNA leaders.

If there is a functional link between archaeal/bacterial HARP and RNA ligase (Rnl3), coordinate gene expression is likely under specific stress conditions; we would expect both (t)RNA processing and ligation activities to be present following co-immunoprecipitation of either the RNA ligase or HARP. Appending short 5′ leaders to C/D box RNAs that are substrates for the archaeal RNA ligase *in vitro* (e.g., sR31, sR42) (25) would provide candidate C/D box precursors for tests with HARP. Such assays, together with biochemical fractionation, would also permit the identification of other cofactors that might enable HARP to process C/D box precursor RNAs. Similarly, we could assess whether 5′-adenylated pre-tRNAs are cleaved by HARP.

Why does nature not use the RNA ligase together with the RNP form of RNase P, which is clearly more widespread? Pre-tRNA recognition by both the RNP (4) and PRORP (33) forms entails binding the invariant and ubiquitous tRNA elbow (specifically, the G19-C56 base pair) to license cleavage. While the structure of HARP (8, 10, 16, 34, 35) shows how it uses its metallonuclease domain in the context of an oligomeric assembly to bind and cleave pre-tRNAs, a somewhat atypical readout of the tRNA elbow suggests that this feature may not be as inviolable as observed with the RNP form. If such flexibility exists with HARP, it will help understand how C/D box precursor RNAs (which lack the signature attributes in pre-tRNAs) could be accommodated and processed.

How do we account for the small fraction of bacteria/archaea that have HARP or ligase but not both? Investigation of the structure and genomic organization of C/D box RNA families across six *Pyrobaculum* species showed that less than two-thirds of the target RNA sites were conserved and that the role of the C/D box RNAs is pliable even among related species (36). If there is no uniform need for processing and circularizing the same set of C/D box RNAs even within a genus, it could help rationalize the patchy phylogenetic distribution of HARP and RNA ligase (Fig. 1).

Analysis of the large, growing cache of genomic data has helped unveil new biological phenomena and provided a toehold for understanding the molecular mechanisms that underlie the diversity of life. Here, we have highlighted the value of this approach to gain an evolutionary perspective on the different guises of RNase P, whose emergence and durability remain an enigma.

## SUPPLEMENTAL MATERIAL

Supplemental material mbio.00449-25-s0001.pdf Description of methods, two supplemental figures, and the caption for Table S1.

Table S1 mbio.00449-25-s0002.xlsx Distribution of HARP and RNA ligase.

ASM does not own the copyrights to Supplemental Material that may be linked to, or accessed through, an article. The authors have granted ASM a non-exclusive, world-wide license to publish the Supplemental Material files. Please contact the corresponding author directly for reuse.
