# Supplementary material for: An RNA ligase partner for the prokaryotic protein-only RNase P: insights into the functional diversity of RNase P from genome mining
Source: mBio. 2025 Apr 29;16(6):e00449-25. doi: 10.1128/mbio.00449-25 (PMC12153264; doi:10.1128/mbio.00449-25)
Supplement: Supplemental material — Description of methods, two supplemental figures, and the caption for Table S1. [file mbio.00449-25-s0001.pdf]

SUPPLEMENTARY MATERIAL

An RNA ligase partner for the prokaryotic protein-only RNase P: insights into the functional diversity of RNase P from genome mining

Rekha Seshadri,[1] and Venkat Gopalan[2,3]

[1]DOE Joint Genome Institute, Lawrence Berkeley National Laboratory, Berkeley, CA 94720, USA

[2]Department of Chemistry and Biochemistry, The Ohio State University, Columbus, OH 43210, USA

[3]Center for RNA Biology, The Ohio State University, Columbus, OH 43210, USA

Correspondence: rseshadri@lbl.gov and gopalan.5@osu.edu

METHODS

Genomes encoding prokaryotic protein-only RNase P (Homolog of Aquifex RNase P or HARP) were identified from the Integrated Microbial Genomes (IMG/M) database (1) based on the presence of PF08745 (PINc domain ribonuclease). For tree building, universally conserved single copy marker protein sequences of the signal recognition particle GTPase (COG0541) were retrieved from these HARP-positive genomes. Alignments of the nonredundant set of markers was performed using MAFFT (2) and maximum likelihood trees were inferred using IQ-Tree (3) using default parameters. The tree (Figure 1, main text) was visualized and annotated using iToL(4). For genome comparisons, 446 HARP-negative genomes were selected from the same taxonomic order as HARP-positive organisms, avoiding redundancies and low-quality genomes. Pair-wise comparisons of gene counts for individual Pfams and Tigrfams between members of HARP-positive (totaling 493) and HARP-negative (totaling 446) sets of archaeal genomes were performed using the IMG/M system which employs a nonparametric Mann-Whitney U-test with Benjamini-Hochberg error correction (5). In both analyses, the RNA ligase was the top enriched function in the HARP-positive set besides HARP itself. The next four significantly enriched functions in the HARP-positive group (by ascending FDR adjusted P-value) include the THUMP domain (PF02926), ADP-specific phosphofructokinase/glucokinase conserved region (PF04587), RNA 3'-terminal phosphate cyclase (RTC) insert domain (PF05189), and tRNA methyltransferase 5 (Trm5) N-terminal domain (PF18093).

SUPPLEMENTARY TABLE LEGENDS

Table S1. List of archaeal/bacterial HARP-positive genomes along with various metadata.

SUPPLEMENTARY FIGURES

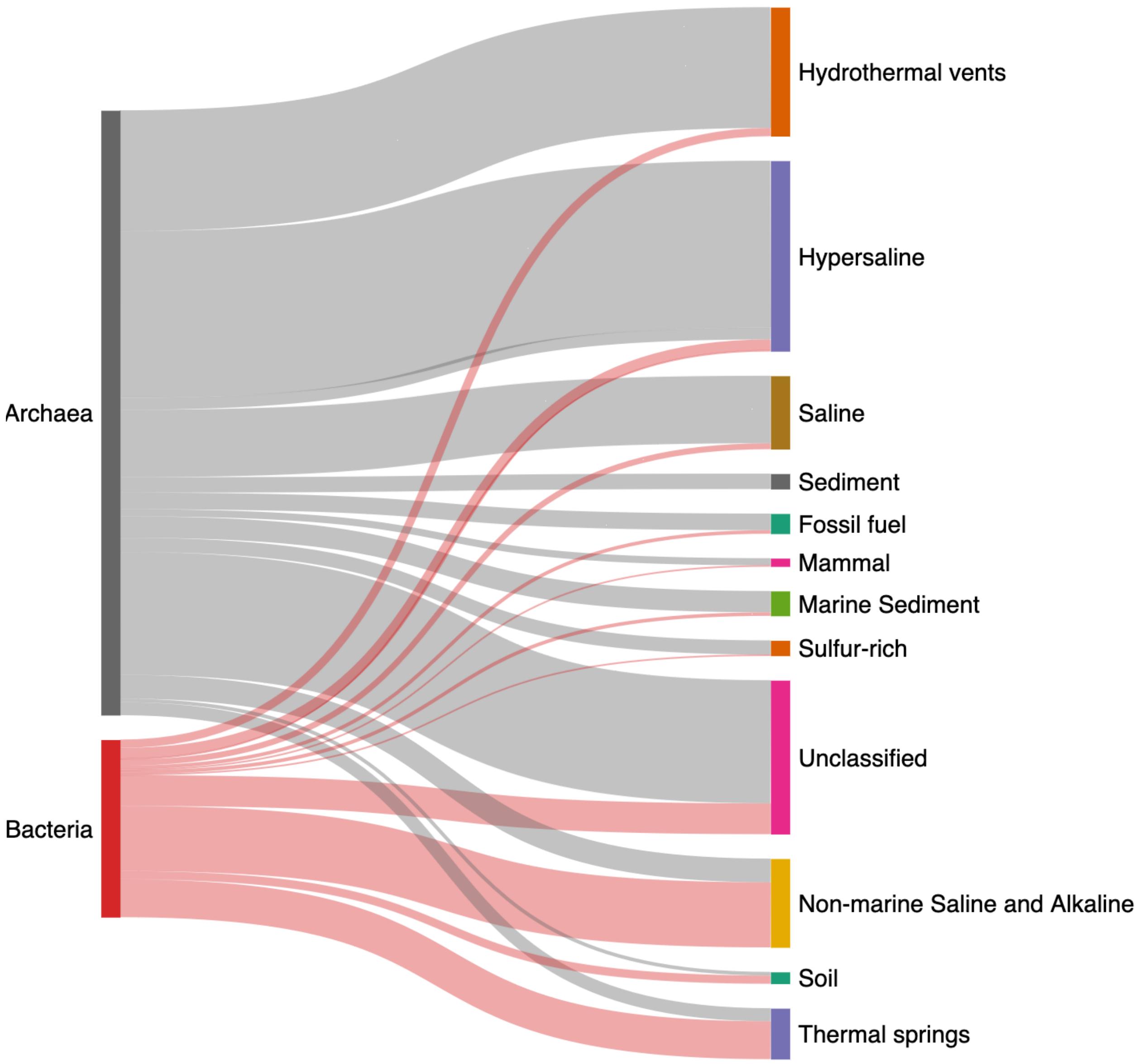

Figure S1. HARP-positive bacterial and archaeal isolate genomes derive from organisms in harsh or extreme habitats. The category "unclassified" includes those with unknown provenance as well as engineered environments (e.g., bioreactors, wastewater). Habitat information was manually curated or sourced from either GOLD (6) or BacDive (7). Diagram was created using SankeyMATIC.

A

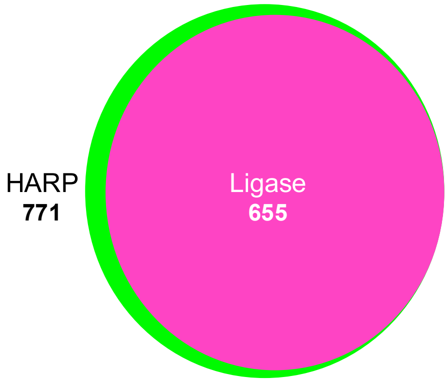

B

Taxa	HARP+	Ligase+	Co-localized
**Archaea**	**573**	**501**	**163**
**Class Halobacteriota**	**394**	**323**	**147**
Halobacteriales	240	169	146
Methanosarcinales	137	137	
Archaeoglobales	10	10	
Methanotrichales	3	3	
Methanonatronarchaeales	3	3	
JAHENH01	1	1	1
**Class Methanobacteriota_B**	**86**	**85**	**6**
Thermococcales	86	85	6
**Class Methanobacteriota**	**80**	**80**	**1**
Methanobacteriales	40	40	
Methanococcales	37	37	1
Methanopyrales	3	3	
**Class Thermoproteota**	**13**	**13**	**9**
Sulfolobales	13	13	9
**Bacteria**	**178**	**164**	**127**
**Class Pseudomonadota**	**115**	**115**	**96**
Ectothiorhodospirales	74	74	61
Nitrococcales	17	17	11
Methylococcales	11	11	11
Nitrosococcales	7	7	7
Thiohalorhabdales	2	2	2
SLND01	2	2	2
DSM-100275	1	1	1
Burkholderiales	1	1	1
**Class Verrucomicrobiota**	**18**	**18**	**18**
Methylacidiphilales	18	18	18
**Class Aquificota**	**15**	**9**	**5**
Aquificales	15	9	5
**Class Desulfobacterota**	**13**	**13**	**2**
Thermodesulfobacteriales	12	12	1
Desulfovibrionales	1	1	1
**Class Nitrospirota**	**9**	**1**	**1**
Thermodesulfovibrionales	9	1	1
**Class Planctomycetota**	**8**	**8**	**5**
Brocadiales	8	8	5

Figure S2. Enumeration and taxonomic distribution of HARP- and RNA ligase-encoding genomes. RNA-ligase positive genomes are all HARP positive.

REFERENCES

1. Chen IA, Chu K, Palaniappan K, Ratner A, Huang J, Huntemann M, Hajek P, Ritter SJ, Webb C, Wu D, Varghese NJ, Reddy TBK, Mukherjee S, Ovchinnikova G, Nolan M, Seshadri R, Roux S, Visel A, Woyke T, Eloe-Fadrosh EA, Kyrpides NC, Ivanova NN. 2023. The IMG/M data management and analysis system v.7: content updates and new features. *Nucleic Acids Res* 51:D723-D732.
2. Katoh K, Rozewicki J, Yamada KD. 2019. MAFFT online service: multiple sequence alignment, interactive sequence choice and visualization. *Brief Bioinform* 20:1160-1166.
3. Trifinopoulos J, Nguyen LT, von Haeseler A, Minh BQ. 2016. W-IQ-TREE: a fast online phylogenetic tool for maximum likelihood analysis. *Nucleic Acids Res* 44:W232-5.
4. Letunic I, Bork P. 2024. Interactive Tree of Life (iTOL) v6: recent updates to the phylogenetic tree display and annotation tool. *Nucleic Acids Res* 52:W78-W82.
5. Chen IA, Chu K, Palaniappan K, Pillay M, Ratner A, Huang J, Huntemann M, Varghese N, White JR, Seshadri R, Smirnova T, Kirton E, Jungbluth SP, Woyke T, Eloe-Fadrosh EA, Ivanova NN, Kyrpides NC. 2019. IMG/M v.5.0: an integrated data management and comparative analysis system for microbial genomes and microbiomes. *Nucleic Acids Res* 47:D666-D677.
6. Mukherjee S, Stamatis D, Li CT, Ovchinnikova G, Bertsch J, Sundaramurthi JC, Kandimalla M, Nicolopoulos PA, Favognano A, Chen IA, Kyrpides NC, Reddy TBK. 2023. Twenty-five years of Genomes OnLine Database (GOLD): data updates and new features in v.9. *Nucleic Acids Res* 51:D957-D963.
7. Reimer LC, Sarda Carbasse J, Koblitz J, Ebeling C, Podstawka A, Overmann J. 2022. BacDive in 2022: the knowledge base for standardized bacterial and archaeal data. *Nucleic Acids Res* 50:D741-D746.
